# Microbial strategies for antagonizing Toll-like-Receptor signal transduction

**DOI:** 10.1016/j.coi.2014.12.011

**Published:** 2015-01-20

**Authors:** Charles V. Rosadini, Jonathan C. Kagan

**Affiliations:** 1 Harvard Medical School and Division of Gastroenterology, Boston Children's Hospital, Boston, MA 02115, USA

## Abstract

Within a few years of the discovery of Toll-like Receptors (TLRs) and their role in innate immunity, viral and bacterial proteins were recognized to antagonize TLR signal transduction. Since then, as TLR signaling networks were unraveled, microbial systems have been discovered that target nearly every component within these pathways. However, recent findings as well as some notable exceptions promote the idea that more of these systems have yet to be discovered. For example, we know very little about microbial systems for directly targeting non-cytoplasmic portions of TLR signaling pathways, i.e. the ligand interacting portions of the receptor itself. In this review, we compare and contrast strategies by which bacteria and viruses antagonize TLR signaling networks to identify potential areas for future research.

## Introduction

Innate immunity is mediated by specialized proteins called pattern recognition receptors that sense microbial invaders and guide our immune systems to eradicate infections. These receptors detect pathogen associated molecular patterns (PAMPs), which are structures common to many microbial species, such as viral nucleic acids or bacterial lipopolysaccharides (LPS). The first identified and most studied group of these receptors, Toll-like-Receptors (TLRs), are displayed at the cell surface and within endosomal compartments where they act as molecular sentinels to detect invading microbes. Upon recognition of a pathogen, in either infected or non-infected cells, TLRs initiate a signaling cascade that leads to expression and release of pro-inflammatory cytokines, chemokines, and Type-I interferons. Cytokine and interferon expression leads to recruitment and activation of immune cells to promote clearance of the infectious agent, but also stimulates expression of genes to block entry and/or replication of microbes. This elegant system is adapted to detect and eliminate almost any threat. However, pathogenic microbes are masters of evading host innate immunity and have evolved a multitude of mechanisms for preventing the antimicrobial activities of TLR signaling pathways. Microbial manipulation of host TLR signaling comes in the form of three main strategies: 1) directly antagonize signaling components 2) avoiding detection by altering their PAMPs to be less immunogenic 3) disrupting cellular organelles/induce cell death. Here we will focus our discussion on direct antagonism approaches.

**Figure 1:**
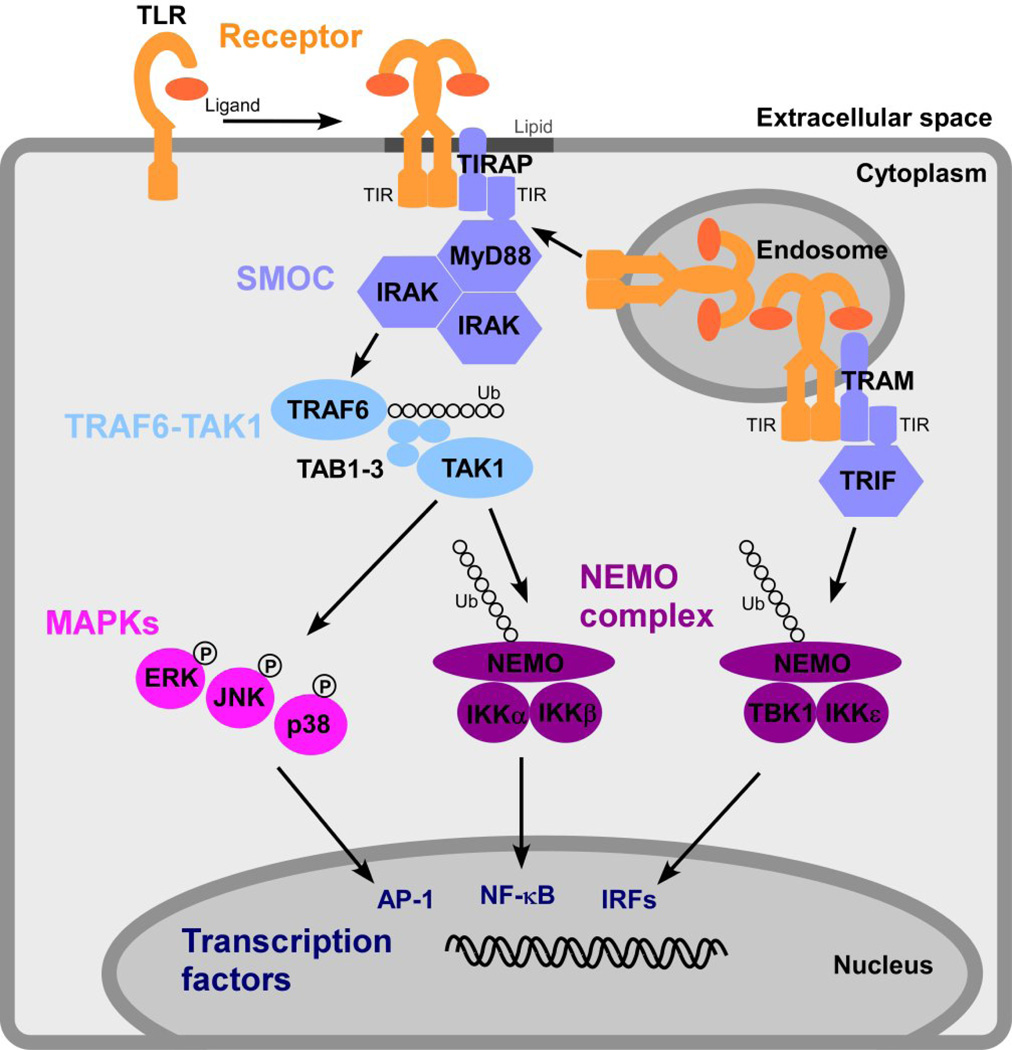
Modules of TLR signaling. The receptor module: binding of ligands to TLRs at the plasma membrane or within endosomes, receptor dimerization and migration to subcellular signaling sites including specific lipid rich regions within the membrane. Supramolecular Organizing Center (SMOC): TLRs interact with intracellular sorting adaptor proteins, TIRAP and TRAM, which recruit signaling adaptors MyD88 and TRIF, respectively. Adaptor proteins and TLRs interact via TIR domains. Signaling adaptors trigger formation of SMOCs such as the myddosome which consists of TIRAP, MyD88, and IRAK kinases. TRAF6 and TAK1 complex: Myddosome formation activates the E3 ubiquitin (Ub) ligase TRAF6 which ubiquitinates itself and recruits TAB proteins. TAB proteins activate the MAP3K, TAK1. Mitogen Activated Protein Kinase (MAPK)s: TAK1 phosphorylates proteins of the MAPK cascade resulting in phosphorylation and activation of MAPKs, ERK, JNK and p38. NEMO complex: Ubiquitinated NEMO is required for IKKα/IKKβ to be activated by TAK1 downstream of Myd88 signaling, but also to promote activation of TBK1 and IKKε downstream of signaling through TRIF. Transcription factors: Transcription factor activation involves activation and nuclear translocation of AP-1 (through MAPKs), NF-κB, through IKKα/IKKβ, and the IRFs through TBK1, IKKε, and in certain circumstances IKKα. Once in the nucleus transcription factors induce expression of proinflammatory cytokines, chemokines, and interferons.

An abundance of viral proteins are known to antagonize TLR signaling at almost every signaling step (reviewed [1]), from blocking signaling intermediates to inhibition of downstream transcription factor activation. While much less is understood about how bacteria block TLR pathways, recent work in this area suggests that they too antagonize several steps of these pathways. We postulated that by surveying the known systems utilized by viruses and bacteria to block TLR signaling, patterns would emerge that would allow us to predict where future research might be most productive. Microbial strategies were organized by the step, or the module, of TLR receptor pathways that they antagonize (Table 1). The receptor module (Fig.1) consists of binding of ligands by various TLRs (reviewed [2]), receptor dimerization, and recruitment of the receptor to subcellular signaling sites such as phosphatidylinositol 4,5-bisphosphate (PIP2) rich regions of the plasma membrane, or phosphatidylinositol 3-phosphate (PI3P) rich endosomes [3, 4]. Once the receptor has moved to signaling sites, it can associate with intracellular sorting adaptor proteins, which by virtue of their association with lipids such as PIP2 and PI3P, are prepositioned on specific organelles to detect activated TLRs. Sorting adaptors, TIRAP and TRAM, promote recruitment of signaling adaptors, MyD88 and TRIF, respectively [5], which are thought to trigger the formation of higher order filamentous structures called Supramolecular Organizing Centers (SMOC) (Fig.1) (Kagan *et al*, In Press). SMOCs are multiprotein complexes that have been proposed to serve as organizing centers that coordinate the multitude of cellular responses to microbial infections and cytokines (Kagan *et al*, In Press). The best-characterized SMOC is that formed between the aforementioned TIRAP and MyD88 adaptors and IRAK kinases. This SMOC has been dubbed the myddosome [4, 6, 7]. Myddosome formation activates a signaling module consisting of an E3 ubiquitin ligase called TRAF6 and the TAK1 complex (Fig.1). TAK1 phosphorylates proteins leading to activation of two signaling modules: the Mitogen Activated Protein Kinases (MAPKs) and the NEMO complex (Fig.1). Signal transduction through MAPKs or the NEMO complex results in the final signaling module: activation and nuclear translocation of transcription factors (Fig.1), such as AP-1 and nuclear factor κB (NF-κB), respectively. In addition, the NEMO complex can also activate transcription factors of the interferon regulatory factor (IRF) family (reviewed [2, 8]). Below we will describe recent findings of microbial systems to antagonize each TLR signaling module.

## The SMOC

SMOC formation immediately follows receptor activation and is initiated by interaction between TLRs and sorting adaptor proteins found on specific organelles within the cell. This interaction occurs between the Toll/IL-1 Receptor/Resistance (TIR) domains present on both TLRs and adaptor proteins [9]. Sorting adaptors recruit signaling adaptors to promote formation of SMOCs, such as the myddosome [4, 5]. The importance of the TLR-induced SMOCs in signal transduction can be revealed by the various pathogenic proteins that have been proposed to counteract the functions of the TIR domain containing adaptors. Microbial systems for antagonizing this signaling module mostly target adaptor proteins and are distributed fairly equally between viruses and bacteria. However, viruses and bacteria tend to rely on their own unique mechanisms of antagonism. For example, viruses heavily favor degrading these adaptor proteins, such as the 3C protease of coxsackievirus B that cleaves TRIF [10], where bacteria often use molecular mimicry by producing proteins that interfere with adaptor protein aggregation. Briefly, bacteria use elaborate secretion systems to inject proteins called effectors into host cells. Some of these effectors contain TIR domains that interact with TIR domains of MyD88, TIRAP, TRIF, TRAM as well as the TLRs. TIR domain containing proteins of bacteria have been reviewed recently [9], however new findings of these proteins in *Yersinia pestis* and *Staphylococcus aureus* demonstrate that this field is rapidly growing and likely more TIR domain containing proteins that can block TLR signaling will emerge [11, 12]. Interestingly, the first protein found to interfere with adaptors to inhibit TLR signaling, A46R of Vaccinia virus (VACV), functions similar to the bacterial TIR domain-containing proteins. A46R binds to MyD88, TIRAP, TRIF, TRAM and TLR4, and can prevent signal transduction [13, 14]. This, however, is not the only example of overlap between microbial strategies for blocking adaptor proteins. In fact, an effector called TcpB from *Brucella spp.* binds TIRAP and induces its ubiquitination and subsequent degradation [15]. This mechanism is reminiscent of the actions of herpes simplex virus (HSV-1) immediate early protein ICP0 that targets MyD88 and TIRAP for degradation [16]. Interestingly, the degree of mimicry of TcpB for TIRAP is remarkable, in that (like TIRAP) it contains a TIR domain and a domain that binds to the plasma membrane via interactions with PIP2. Prior to inducing TIRAP degradation, TcpB binds to TIRAP and prevents its interaction with TLR4 [17, 18]. Therefore, TcpB represents a unique example where a single protein binds and physically blocks but also promotes degradation of adaptors. Unlike ICP0, which potentially targets TIRAP via its E3 ubiquitin ligase activity, it remains unclear how TcpB induces TIRAP degradation. Future study of the function of this protein and its interactions with TIRAP will lead to greater understanding of the earliest modules of TLR signaling. Overall, findings of bacterial proteins that target the adaptors will likely continue. Intriguingly, while several microbial proteins engage myddosome components through TIR-TIR interactions, only one protein is known to antagonize kinases within the myddosome. The A52R protein of VACV binds IRAK2, a myddosome component, to inhibit signaling [19]. Future research will be needed to determine if other microbes utilize similar strategies to inhibit TLR signaling at the module of SMOC assembly or function.

## TRAF6 and TAK1 complex

Myddosome formation involves the recruitment of IRAK family kinases, which in turn activate a module consisting of the E3 ubiquitin ligase TRAF6 and the MAP3K, TAK1. TRAF6 polyubiquitinates itself and recruits TAK1 regulatory components called TAK1-binding protein (TAB) 1–3. Binding of TAB2 and TAB3 to ubiquitin chains made by TRAF6 is required to activate TAK1 [20]. Active TAK1 phosphorylates proteins of the MAPK cascade and also the NEMO complex, leading to their activation (discussed in detail below). In the last few years, several discoveries of both viral and bacterial mechanisms for antagonizing the TRAF6 and TAK1 complex have emerged. First, the kinase U_S_3 of HSV-1 was shown to reduce levels of polyubiquitination on TRAF6, resulting in inhibition of TLR2 signaling during viral infection [21]. Inhibition of TLR2 signaling by U_S_3 is dependent on its kinase activity and found to be downstream of MyD88. However, how this protein affects TRAF6 ubiquitination remains an open question [21]. Next, recent work on Epstein-Barr virus protein BPLF1 suggests that this protein is a deubiquitinase that also inhibits ubiquitination of TRAF6 [22, 23]. Furthermore, OspI of *Shigella flexneri* is an example of a bacterial protein that inhibits TRAF6 polyubiquitination [24]. However, this enzyme uses a unique approach by deamidating a glutamine residue in the E2 ubiquitin conjugating enzyme UBC13, which is required for ubiquitination of TRAF6 [24]. Enzymes that target activation of the TAK1-TAB1–3 complex downstream of TRAF6 were also described, including NleE from Enteropathogenic *Escherichia coli* (EPEC) and YopJ from *Yersinia pestis* [25, 26]. NleE is a methyltransferase that targets critical zinc coordinating cysteine residues within zinc-finger domains of TAB2 and TAB3. This novel mechanism results in loss of ubiquitin binding activity of TAB2 and TAB3, preventing activation of TAK1 and subsequently, NF-κB [25]. In contrast, YopJ inhibits TAK1 kinase activity by acetylating key residues in its active site [26]. In this way YopJ blocks TAK1-dependent activation of MAP2Ks and inhibitor of NF-κB kinases (IKKs). Interestingly, unlike what has been found for targeting many of the other modules of TLR signaling, the mechanisms for targeting this module are diverse and dissimilar between viruses and bacteria. Perhaps future research will uncover how common each of these mechanisms are across a broad range of pathogens. On the other hand, with so few examples described, potentially other fascinating mechanisms for blocking this module of signaling will be discovered.

## MAPK Cascade

The MAPK cascade is a phospho-relay system that signals through ERK, JNK and p38 to activate the transcription factor AP-1. Previously, only bacterial systems were known to block MAPK cascades (reviewed [27]). However, recent research revealed viral proteins capable of targeting MAPKs, including VP24 of Ebola virus, which inhibits interferon-β (IFN-β) stimulated JAK-STAT signaling by blocking phosphorylation of p38 [28]. Furthermore, the surface antigen of Hepatitis B virus (HBsAg) blocks TLR2 signaling by inhibiting phosphorylation of JNK-1/2 and c-Jun, although the mechanism for this inhibition remains unclear [29]. Together these examples not only demonstrate that viruses have the ability to block TLR signaling at this module, but also suggest that viruses target select MAPKs. The advantage of targeting select MAPKs may be to ensure maintenance of other MAPK-dependent pathways that promote survival of the infected cell.

## NEMO complex

Downstream of MyD88 and TAK1 is a signaling module governed by the protein NF-κB essential modulator (NEMO). NEMO controls the activation of IKKs, such as IKKα and IKKβ. NEMO binds non-degradative polyubiquitin chains that act as a scaffold for TAK1 phosphorylation of IKKα/IKKβ [20]. IKKα/IKKβ, in turn phosphorylates the inhibitor of NF-κB, IκB, promoting its ubiquitination and subsequent degradation, releasing NF-κB so it can translocate to the nucleus. In the case of endosomal TLRs, IKKα can also promote IRF7 activation. Alternatively, signaling downstream of TRIF leads to NEMO complex activation, however this results in recruitment and activation of TBK1/IKKε and their accessories TANK, SINTBAD, and NAP1. These proteins function to activate the transcription factor IRF3, which is a master regulator of interferon expression [30].

Diverse microbial strategies are used to target the NEMO complex including degrading, binding, and even deubiquitinating host enzymes and accessory proteins. Recent discoveries revealed that the 3C proteases from hepatitis A virus and foot-and-mouth disease virus can inhibit TLR signaling by cleaving NEMO [31, 32]. Additionally, C-protein of Sendai virus blocks TLR7 and 9 signaling by binding IKKα and inhibiting phosphorylation of IRF7 [33]. Furthermore, BPFL1 of Epstein-Barr virus blocks signaling by deubiquitinating IKKα and NEMO [23]. Interestingly, although a limited number of bacterial proteins are known to target this module, one such effector, IpaH9.8 of *S. flexneri* utilizes a strategy similar to viruses by promoting the degradation of NEMO. This example of bacteria using a strategy similar to that of viruses promotes the idea that knowledge of viral strategies can be used as a framework for future investigation of novel bacteria proteins that antagonize this signaling module.

## Transcription Factors

Transcription factor activation involves nuclear translocation of AP-1, NF-κB and the IRFs, which lead to transcriptional responses and the production of pro-inflammatory cytokines, chemokines, and interferons. Targeting the transcription factor module is a widely used strategy for viruses. Viral mechanisms for targeting this module include degrading and mimicking transcription factors and have been extensively reviewed elsewhere [34, 35]; therefore we will focus on a few recent findings that highlight some of their tactics. First, HSV-1 and rotavirus prevent NF-κB translocation into the nucleus by stabilizing or preventing the degradation of IκB [36, 37]. This tactic is also used by VACV, whose protein A49 inhibits this signaling module by binding and inhibiting the activity of β-TrCP, an E3 ligase required for ubiquitination and degradation of IκB [38]. A second mechanism is that used by ORF47 of varicella-zoster virus and U_S_3 of HSV-1, which phosphorylate IRF3 to block its proper activation by host kinases [39, 40]. Recently, U_S_3 was also shown to hyperphosphorylate NF-κB, blocking its activation [41]. Thirdly, some viruses bind co-activators such as CBP and p300 in the nucleus, an activity that prevents transcription factors from reaching their target genes. This method is used by herpesvirus proteins including ICP0 of HSV-1 and vIRF-1 of Kaposi's sarcoma-associated herpesvirus (KSHV) [42, 43]. Recent data suggests that VP16 of HSV-1 is also capable of binding to CBP in the nucleus [44].

Despite the abundance of viral species that block this module, only a few bacterial species are known to possess this ability. These include enterohemorrhagic *Escherichia coli* (EHEC), EPEC, and *S. flexneri*. EHEC and EPEC encode effectors NleH1 and NleH2 that block NF-κB by binding to NF-κB subunit, RPS3 [45, 46]. NleH1 but not NleH2 was shown to inhibit nuclear translocation of RPS3, however both NleH1 and NleH2 have also been implicated in preventing ubiquitination and degradation of IκB [46]. EPEC also encodes NleC and NleD which are zinc metalloproteases that cleave the RelA subunit of NF-κB to prevent its activation [47–50]. Two other EPEC proteins, NleB and NleE, also block NF-κB translocation [51]. The targets of NleB and NleE are not known, however their mechanisms are likely different as NleB blocks signaling in response to stimulation of cells with TNFα but not IL-1β, whereas, NleE blocks both TNFα and IL-1β stimulated responses. *S. flexneri* encodes OspZ, which is interchangeable with the C-terminus of NleE, and thus likely acts via a similar mechanism [51]. A second *S. flexneri* encoded effector, OspG, acts similar to VACV A49 discussed above. OspG prevents degradation of IκB by binding and interfering with the E2 ubiquitin-conjugating enzyme, UbcH5b [52, 53]. This example of overlap between bacterial and viral tactics reinforces the idea that future investigation into bacterial proteins that antagonize TLR signaling can be guided by our vast knowledge of viral antagonization mechanisms.

## Targeting multiple modules

A common tactic utilized by viruses and bacteria is to attack several modules of TLR signaling simultaneously. For example, VACV expresses N1L to block IKKs but also expresses A46R and A52R to target adaptor proteins and IRAK kinases [13, 19, 54], respectively, whereas *S. flexneri* expresses OspF which blocks MAPKs, OspI which blocks TRAF6, and IpaH9.8 which promotes degradation of NEMO. Other examples include *Salmonella* spp. which express TlpA, a TIR domain containing protein that interferes with adaptor proteins [55], and SpvC, a phosphothreonine lyase that irreversibly modifies MAP kinases [56, 57]. It is also common for viruses and bacteria to utilize a single protein for blocking more than one module of TLR signaling. Examples include: BPLF1 of Epstein-Barr virus which deubiquitinates TRAF6, IKKα, and NEMO [22, 23], ICP0 of HSV-1 which promotes degradation of TIRAP and MyD88 but also binds and sequesters IRF3 in the nucleus [16, 42], and YopJ of *Yersinia spp*., which acetylates several MAPKs and IKKβ [26, 58], effectively eliminating MyD88-dependent signaling through both the MAPK cascade and the NEMO complex. This strategy is not surprising for viruses as they express a limited number of proteins and thus often utilize a single protein to perform diverse functions. It is more intriguing however for bacteria, which can inject dozens of proteins into host cells [59]. However, for some species that inject only a small number of proteins into host cells, such as *Y. pestis* which only secretes 6 known effectors [60], this strategy suggests a need for fine tuning of their existing proteins to target multiple host factors. Overall, the popularity of these strategies suggests that if a virus or bacterium is known to antagonize TLR signaling at one module, it will likely be found to target other modules.

## Receptors

In our search for microbial systems that antagonize specific components of TLR signaling networks, we find that viruses are not known to antagonize TLR signaling at the module of the receptor. Perhaps this is due to a lack of effective tools, such as antibodies, for detecting changes in TLRs on the surface of cells during infections. We also note that this module of TLR signaling is the least understood. Assays to monitor inducible (microbe-triggered) interactions between TLRs and their ligands or co-receptors are very much lacking. Despite these technical challenges, it is possible that viruses may not interfere with TLR detection directly. One reason to consider this possibility is that it may be more efficient for them to target downstream signaling components that are used by multiple innate immune pathways than to antagonize individual receptors. Nevertheless, there are some viral strategies that could antagonize TLRs. For example, viral proteins in both human T-cell leukemia virus type 1 (p30) and HSV-1 (U_S_3) can promote down-regulation of TLR4 and TLR3, respectively [61, 62]. It remains unclear how common this strategy is for other viruses, and whether the down-regulation observed is sufficient to functionally inactivate the signaling pathway.

Despite the challenges of investigating the receptor module, recent discoveries have revealed that at least two bacterial proteins are capable of binding to TLRs and blocking their function. The secreted staphylococcal superantigen-like proteins (SSLs) SSL3 and SSL4 from *S. aureus* have the ability to interact with the ectodomain of TLR2 [63, 64]. Incubation of mouse or human leukocytes with purified SSL3 blocks TLR2-dependent responses to purified TLR2 ligands as well as heat killed *S. aureus*. However, the ability of *S. aureus* to suppress TLR2 responses in a SSL3/4 dependent manner during *S. aureus* infection has not been demonstrated. Thus, the relevance of this system for virulence and survival of *S. aureus* remains an open question. Interestingly, other SSL proteins of *S. aureus* have been shown to bind and block extracellular immune components on leukocytes such as SSL5 which binds chemokine and anaphylatoxin receptors [65]. Thus, SSLs demonstrate the potential for bacterial proteins to antagonize host immune functions by binding to extracellular components.

Other bacterial strategies also suggest possible mechanisms for direct TLR antagonism, although these strategies have never been directly implicated in antagonizing these receptors. For example, bacteria can secrete proteins to degrade host immune components such as the IgA proteases of *Streptococcus pneumoniae, Haemophilus influenzae*, and *Neisseria spp.*, [66, 67] and also secrete many other proteases that have been linked with virulence and survival of different bacterial species. Interestingly, the cysteine proteases, known as gingipains, from *Porphyromonas gingivalis* have been shown to preferentially cleave CD14, an extracellular co-receptor vital to TLR4 signaling, from phagocytes [68], though the relevance of this to survival and virulence of *Porphyromonas spp.* remains unclear. This example suggests that bacterial proteases have the potential to counteract TLR function and inflammatory responses through direct cleavage of receptor complexes on the surface of host cells. Perhaps future investigation of viral or bacterial proteins and their interaction with TLRs will reveal novel systems for targeting the receptor module.

## Perspective

Recent years have seen an explosion in findings of bacterial and viral systems to antagonize TLR signaling. Here we have compiled a wide-ranging list of these known strategies with the intent of providing insight into where future research will be best applied. Notably, we have identified gaps in our knowledge, such as the lack of systems for targeting the receptor module, which present some interesting challenges for the field. Overall, our findings indicate that viruses and bacteria have evolved similar mechanisms for antagonizing TLR signaling. This evolutionary convergence of strategies suggests that the information presented here can be used to guide future discovery of novel microbial factors that antagonize TLR signaling, both viral and bacterial.

## Figures and Tables

**Table. 1:** 

Module Targeted	Microbe	Protein	Mode of antagonization	Citation
**Receptor**				
	*Staphylococcus aureus*	SSL3, SSL4	Bind TLR2 ectodomain	[63, 64]
**SMOC**				
	African swine fever virus	ORF I329L	Possibly targets TRIF	[69]
	Coxsackievirus	3C	Degrades TRIF	[10]
	Enterovirus 68	3C	Cleaves TRIF	[70]
	Hepatitis C virus (HCV)	NS3-4A	Cleaves TRIF	[71]
	HCV	NS5A	Binds MyD88	[72]
	HSV-1	ICP0	Promotes degradation of Myd88 and TIRAP	[16]
	KSHV	RTA	Degrades TRIF	[73]
	Vaccinia virus (VACV)	A46R	Binds TIRAP, TRAM, MyD88, TRIF, TLR4	[13, 14]
	VACV	A52R	Binds IRAK2	[19]
	Brucella spp.	BtpB	Probable TIR domain containing	[74]
	*Brucella melitensis*	TcpB	Blocks TIR-TIR interactions and promotes degradation of MyD88 and TIRAP	[75]
	*Escherichia coli*	TcpC	Blocks TIR-TIR interactions	[75]
	*S. aureus*	TirS	Blocks TIR-TIR interactions	[12]
	*Salmonella enterica serovar Enteritidis*	*TlpA*	Blocks TIR-TIR interactions	[55]
	*Yersinia pestis*	YpTdp	Blocks TIR-TIR interactions	[11]
**TRAF6/TAK1**				
	Herpes simplex virus (HSV-1)	Us3	Decreases levels of TRAF6 ployubiquitination	[21]
	Epstein-Barr virus (EBV)	BPLF1	Deubiquitinates TRAF6	[22, 23]
	*Shigella flexneri*	OspI	Deamidates UBC13	[24]
	Enteropathogenic *E.coli* (EPEC)	NleE	Modifies TAB2 and TAB3	[25]
	Yersinia spp.	YopJ	Acetylates TAK1	[26]
**MAP kinases**				
	Ebola virus	VP24	Blocks phosphorylation of p38	[28]
	Hepatitis B virus	HBsAg	Inhibits phosphorylation of JNK1/2 and c-Jun	[29]
	*Bacillus anthracis*	LF	Degrades MAPKK 1 and 2	[76]
	EPEC	NleC and NleD	Cleaves JNK	[50]
	*Salmonella typhimurium*	AvrA	Acetylates MKK4	[77]
	Salmonella spp.	SpvC	Modifies c-Jun, Erk1/2, and p38	[56, 57]
	*S. flexneri*	OspF	Modifies c-Jun, Erk1/2, and p38	[56, 78]
	*Vibrio parahemolyticus*	VopA	Acetylates MAPKK	[79]
	Yersinia spp.	YopJ	Acetylates MKK6, MKK4, MKK7	[26, 58]
**NEMO complex**				
	EBV	BPLF1	Deubiquitinates NEMO	[23]
	Foot-and-mouth disease virus	3C	Cleaves NEMO	[31]
	Hepatitis A virus	3C	Cleaves NEMO	[32]
	HCV	NS3	Binds TBK1	[80]
	HSV-1	γ34.5	Binds TBK1	[81]
	Mouse hepatitis virus A59	NSp3 (PLP2 domain)	Deubiquitinates TBK1	[82]
	Sendai virus	C-protein	Binds IKKα	[33]
	VACV	B14R	Binds IKKβ	[83]
	VACV	C6	Binds TANK, SINTBAD, or NAP1	[84]
	VACV	N1L	Associates with IKK complex and TBK1	[54]
	VACV	K7R	Binds DDX3	[85]
	*S. flexneri*	IpaH9.8	Promotes NEMO degradation	[86]
	Yersinia spp.	YopJ/P	Acetylates IKKβ	[87]
**Transcription factors**				
	Classical Swine Fever Virus	Npro	Interacts with IRF7	[88]
	EBV	BPLF1	Deubiquitinates IκBα	[23]
	HIV	Vpr, Vif	Degrade IRF3	[89, 90]
	HIV	Vpu	Possibly blocks IRF3 and NF-κB	[90, 91]
	HSV-1	ICP27	Stabilizes IkBa	[36]
	HSV-1	ICP0	Sequesters IRF3- CBP/p300	[42]
	HSV-1	Us3	Phosphorylates IRF3 and NF-kB	[40, 41]
	HSV-1	VP16	Binds CBP in the nucleus	[44]
	KSHV	RTA	Promotes degradation of IRF3 and IRF7	[92]
	KSHV	K-bZIP	Competes for IRF3 binding sites	[93]
	KSHV	vIRF-1	Inhibits IRF3 interaction with CBP and p300	[43]
	KSHV	vIRF3	Binds IRF3	[94]
	Measles virus	C-protein	Unknown function in nucleus	[95]
	Measles virus	V-protein	Binds NF-kB and IRF3	[96]
	Mumps virus	V-protein	Mimics IR3	[97]
	Rotavirus	NSP1	Degrades IRF3, IRF7 or E3 ligase β-TrCP	[37, 98, 99]
	Sendai virus and New castle disease virus	V-protein	Binds active IRF3 and prevents nuclear translocation	[96]
	VACV	A49	Binds and inhibits β-TrCP	[38]
	Varicella-Zoster virus (VZV)	ORF47	Atypically phosphorylates IRF3	[39]
	VZV	ORF61	Ubiquitinates IRF3 and NF-kB	[100, 101]
	EPEC	NleC,NleD	Cleaves NF-κB	[47–50]
	EPEC	NleB, NleE	Inhibits nuclear translocation of NF-kB	[51]
	EPEC and enterohemorrhagic *Escherichia coli* (EHEC)	NleH1, NleH2	Inhibits nuclear translocation of NF-kB	[45, 46]
	*S. flexneri*	OspZ	Inhibits nuclear translocation of NF-kB	[51]
	*S. flexneri*	OspG	Binds and interferes with UbcH5b	[52]

## Highlights

- We assemble a collection of bacterial and viral mechanisms for blocking TLR signaling networks directly.
- We analyze recent findings of novel microbial strategies that inhibit TLR signaling and identify where future research is likely to be most productive.
- We discuss the limited knowledge of microbial systems for antagonizing the very earliest module of TLR signaling: the receptor module.
